# Photodynamic therapy with a novel photosensitizer inhibits BLM-induced pulmonary fibrosis in mice via MRC1-mediated pathway

**DOI:** 10.3389/fphar.2025.1714450

**Published:** 2025-11-27

**Authors:** Minghui Zhu, Nan Wang, Feiyu Zhang, Yumei Rong, Zhenyu Zhao

**Affiliations:** 1 NHC Key Laboratory of Hormones and Development, Tianjin Key Laboratory of Metabolic Diseases, Chu Hsien-I Memorial Hospital & Tianjin Institute of Endocrinology, Tianjin, China; 2 Tianjin Medical University, Tianjin, China; 3 Central Hospital,Tianjin University/The Third Central Hospital of Tianjin, Tianjin, China; 4 Tianjin Key Laboratory of Extracorporeal Life Support for Critical Diseases, Tianjin, China; 5 Artificial Cell Engineering Technology Research Center, Tianjin, China; 6 Tianjin Institute of Hepatobiliary Disease, Tianjin, China

**Keywords:** pulmonary fibrosis, photodynamic therapy, oxidative stress, bleomycin, Mrc1

## Abstract

**Background:**

Pulmonary fibrosis (PF) is a devastating interstitial lung disease with limited therapeutic options, characterized by progressive extracellular matrix deposition and irreversible functional decline. Photodynamic therapy (PDT) represents a promising therapeutic modality, but its application in PF is hindered by the lack of effective and safe photosensitizers. This study aimed to investigate the therapeutic potential and underlying mechanisms of a novel photosensitizer, LD4, in a murine model of PF.

**Methods:**

The anti-fibrotic efficacy of PDT-LD4 was evaluated both *in vitro* using human embryonic lung fibroblasts (HELF) and *in vivo* in a bleomycin (BLM)-induced pulmonary fibrosis model in mice. Mice were randomly allocated into control, model (BLM), pirfenidone (PFD, positive control), and three PDT-LD4 dose groups (60, 120, and 240 μg/kg). Treatments were administered weekly via intratracheal instillation followed by thoracic irradiation (650 nm, 25 J/cm^2^). Assessments included survival rate, histopathology, inflammatory cytokine levels (*IL-1β, IL-6, TNF-α*), oxidative stress markers (*SOD, GSH, MDA, ROS*), collagen deposition (*Collagen I/III* immunohistochemistry, hydroxyproline content), label-free quantitative proteomics, and molecular docking.

**Results:**

PDT-LD4 significantly inhibited HELF proliferation while exhibiting low dark toxicity. In BLM-induced mice, PDT-LD4 markedly improved survival rates and attenuated body weight loss. Histopathological analysis demonstrated that PDT-LD4 substantially reduced inflammatory infiltration and collagen deposition. Mechanistically, PDT-LD4 suppressed pro-inflammatory cytokine levels, alleviated oxidative stress by restoring antioxidant capacity, and inhibited collagen synthesis. Proteomic profiling identified macrophage mannose receptor (*MRC1*) as a differentially expressed protein, whose BLM-induced upregulation was reversed by PDT-LD4, suggesting that the regulation of pulmonary fibrosis (PF) may involve the modulation of *MRC1*. Molecular docking confirmed a stable binding interaction between LD4 and *MRC1*. Importantly, systemic toxicity assessment revealed no significant damage to major organs.

**Conclusion:**

PDT-LD4 exerts anti-fibrotic effects in bleomycin-induced pulmonary fibrosis through multiple mechanisms including anti-inflammation, anti-oxidation and anti-fibrosis, which may be related to the regulation of *MRC1*. Given its proven efficacy and good safety, PDT-LD4 emerges as a promising novel therapeutic strategy and is worthy of further clinical research for pulmonary fibrosis.

## Introduction

Pulmonary Fibrosis (PF) is a chronic disease of unknown etiology characterized by diffuse alveolar inflammation and abnormal pathological proliferation of fibroblasts (Zhao et al., 2023). PF is marked by its insidious onset, progressive worsening, high mortality rate, and an increasing annual incidence (Huang et al., 2013; Mohamed et al., 2024). In recent years, complex etiological factors such as atmospheric pollution and paraquat inhalation have contributed to a rising incidence; however, therapeutic options remain severely limited. Currently, the most effective treatment for PF is lung transplantation. Nevertheless, its widespread application is constrained in many countries, including China, due to donor shortage. Home oxygen therapy can ameliorate hypoxia and improve exercise capacity but does not modify the underlying disease progression. Despite advancing insights into its pathophysiology, pulmonary fibrosis (PF) remains a devastating and relentlessly progressive interstitial lung disease with limited therapeutic options. The current standard of care, primarily comprising anti-fibrotic agents such as pirfenidone and nintedanib (Qiu and Ye, 2025), aims to decelerate disease progression but falls short of achieving disease reversal or offering a cure. The formidable challenges posed by PF—including its complex pathogenesis involving recurrent alveolar epithelial injury, dysregulated fibroblast activation, and excessive extracellular matrix (ECM) deposition—underscore the imperative for developing novel treatment modalities that target these core pathological mechanisms directly.

A hallmark pathological feature of pulmonary fibrosis is the abnormal accumulation of extracellular matrix, among which excessive collagen deposition represents a critical process driving disease progression. Therefore, precise assessment of collagen metabolism and deposition is essential for elucidating the mechanisms of fibrosis and evaluating interventional strategies (Zhang et al., 2025a). An imbalance between oxidative stress and antioxidant defense mechanisms leads to the excessive generation of free radicals (Hao et al., 2024). These radicals attack biomembranes, inducing lipid peroxidation and consequent formation of Malondialdehyde (MDA), which causes cellular damage and exerts significant toxic effects on cells and tissues. Quantifying MDA levels provides a measure of oxidative stress intensity. Superoxide Dismutase (SOD), a commonly used marker of antioxidant status, plays a vital role in maintaining the redox equilibrium. Measuring SOD activity helps further evaluate the antioxidant capacity. Glutathione (GSH) contributes to antioxidant defense by facilitating the decomposition of hydrogen peroxide and inhibiting the formation of hydroxyl radicals.

The macrophage mannose receptor (MRC1, also known as CD206) has emerged as a protein of significant interest in the context of fibrotic diseases, including pulmonary fibrosis. MRC1 is a C-type lectin receptor predominantly expressed on macrophages and is widely recognized as a key marker for anti-inflammatory, pro-fibrotic M2 macrophage polarization. Its role in fibrosis, however, appears complex and context-dependent. A foundational study by von Ehr et al. established a clear antagonistic relationship between *TGF-β* signaling and *MRC1*, demonstrating that *TGF-β1* downregulates *MRC1* transcription in microglia, whereas genetic or pharmacological silencing of TGF-β signaling robustly upregulates it (Von Ehr et al., 2020). This inverse relationship suggests that the observed upregulation of *MRC1* in fibrotic models may reflect an underlying dysregulation of the canonical *TGF-β* signaling pathway, a master driver of fibrosis. Furthermore, emerging evidence links *MRC1* expression directly to pro-fibrotic outcomes. Liu et al. (Liu et al., 2024) recently demonstrated that deficiency in *NLRX1*, a mitochondrial immune regulator, promotes M2 macrophage polarization with concomitant increases in *MRC1* expression and *TGF-β* secretion, ultimately leading to exacerbated renal fibrosis. Crucially, this pro-fibrotic phenotype was driven by a metabolic shift towards enhanced mitochondrial oxidative phosphorylation (OXPHOS), underscoring an intimate link between macrophage immunometabolism, *MRC1* expression, and fibrotic output. These findings position *MRC1* not merely as a passive marker but as a potential functional component within a profibrotic signaling network, making it a novel and compelling target for anti-fibrotic therapy.

Photodynamic therapy (PDT), a well-established modality in oncology and select non-malignant conditions (Shi et al., 2025), presents a compelling and innovative therapeutic paradigm worthy of exploration for PF. The rationale for investigating PDT in PF extends well beyond its documented antimicrobial and anti-inflammatory effects observed in other disease models, and stems from its unique mechanistic action that strategically converges upon several core pathways implicated in fibrogenesis. The classical PDT process involves the administration of a photosensitizer (PS), followed by its selective accumulation in target tissues and subsequent activation by light of a specific wavelength. This activation triggers photochemical reactions that generate cytotoxic reactive oxygen species (ROS) (Li et al., 2025). In the specific context of PF, this controlled, localized ROS burst can be therapeutically harnessed to induce selective apoptosis in hyperactive, apoptosis-resistant fibroblasts and myofibroblasts—the primary effector cells responsible for excessive extracellular matrix (ECM) deposition. This represents a direct cytotoxic approach to eliminating the cellular drivers of fibrosis, a mechanism distinct from and complementary to the mere suppression of inflammation. Furthermore, beyond direct cell killing, the immunomodulatory sequelae of PDT can disrupt the persistent pro-fibrotic signaling milieu and alter cell-cell communication within the fibrotic niche, potentially halting or even reversing the fibrotic cascade. Thus, the fundamental rationale for repurposing PDT for fibrosis lies in its capacity to simultaneously target the aberrant cellular actors, the pathological mechanical and signaling microenvironment, and the dysregulated immune response, offering a multi-pronged intervention against a complex disease process.

We hypothesize that the controlled cytotoxic and immunomodulatory effects of PDT can be strategically harnessed to disrupt the vicious cycle of PF. Specifically, targeting hyperplastic, apoptosis-resistant fibroblasts and myofibroblasts represents a promising strategic application. Furthermore, the ability of PDT to alter the pro-fibrotic microenvironment and attenuate chronic inflammatory signaling could potentially halt or even reverse fibrotic progression. This article aims to elucidate the scientific rationale for repurposing PDT for PF, review pertinent preclinical evidence, discuss potential targeting strategies within the lung, and critically address the challenges and future directions for translating this intriguing concept into a viable clinical intervention. 5,10,15,20-Tetra{4-[(S)-2,6-diamino-hexamide] phenyl} porphyrin (LD4) is a water-soluble, low-toxicity porphyrin derivative developed in our laboratory (Meng et al., 2015). Previous studies have demonstrated the efficacy of LD4-based photodynamic therapy (PDT-LD4) in promoting wound healing and modulating immune responses in infected wounds (Xu et al., 2016; Zhao et al., 2021), showing significant antimicrobial activity. Furthermore, its therapeutic potential has been confirmed in a model of trinitrobenzene sulfonic acid (TNBS)-induced ulcerative colitis (Rong et al., 2021). Building on these findings, which underscore LD4’s robust anti-inflammatory and immunomodulatory properties, we hypothesize that PDT-LD4 could be repurposed to inhibit pulmonary fibrosis. In this study, we investigated PF using the bleomycin-induced mouse model. While this model replicates key pathological features of human PF, such as inflammation, oxidative stress, and collagen deposition, it is primarily used to explore general fibrotic mechanisms rather than the specific etiology of IPF and we aimed to investigate the therapeutic potential and underlying mechanisms of our novel photosensitizer, LD4, in PDT for the treatment of BLM-induced pulmonary fibrosis in mice. We hypothesized that PDT-LD4 would attenuate PF through multi-targeted effects on inflammation, oxidative stress, and collagen deposition. This approach may overcome the limitations of current treatments and establish LD4 as an effective photosensitizer for anti-fibrotic drug development, potentially leading to a novel, high-efficacy, and low-toxicity therapeutic agent for fibrosis.

## Materials and methods

### Drugs and reagents

**FIGURE 1:**
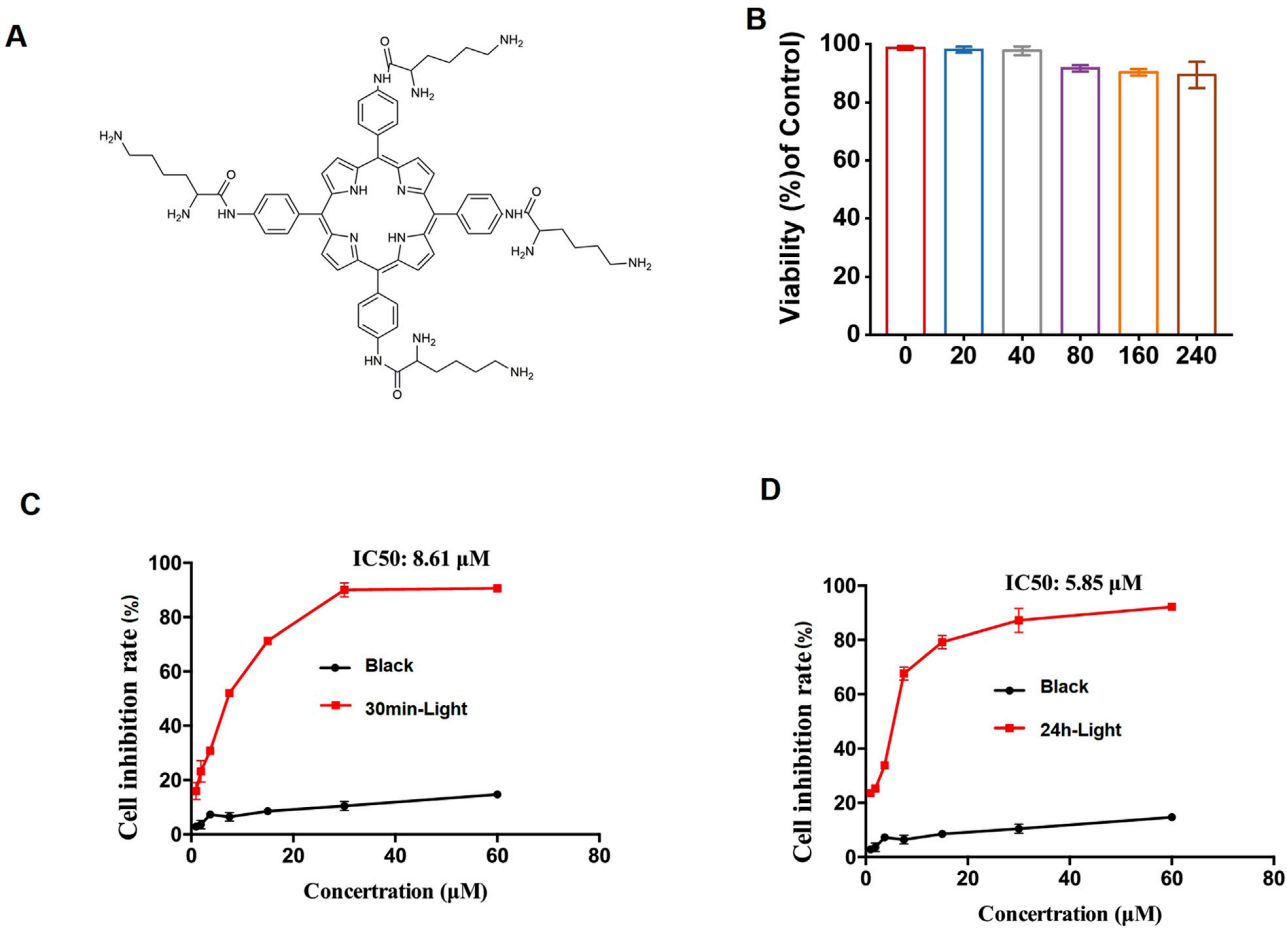
The effect of PDT-LD4 on HELF cells. **(A)** The chemical structure of LD4. **(B)** The MTT method was used to examine the safety of LD4 in HELF cells. The MTT method was used to examine the effects of LD4 on the light reaction and black reaction in the prevention **(C)** and treatment **(D)** of pulmonary fibrosis in the HELF cell model, as well as the safety of the treatment on the HELF cells. Data were expressed as mean ± SD (n = 3 per group).

The photosensitizer LD4 was synthesized and characterized in our laboratory following previously reported procedures (Meng et al., 2015). Its chemical structure is shown in Figure 1A.

### Cell culture

Human Embryonic Lung Fibroblasts (HELF) were maintained in DMEM (Gibco) supplemented with 10% fetal bovine serum (Lonsera, S711-001S) at 37 °C under a humidified atmosphere containing 5% CO_2_. HELF were randomly seeded into 96-well plates. Prior to the MTT assay, the control and model groups were refreshed with DMEM, while other wells were incubated for 24 h in serum-free RPMI-1640 medium containing various concentrations of LD4. The black reaction control group consisted of HELF treated with identical concentrations of LD4 but strictly shielded from light throughout the incubation period to assess the dark toxicity of LD4 itself, independent of photodynamic activation.

### Animals

BALB/c mice (6–8 weeks old) were supplied by BeiJing HFK Bioscience Co., Ltd. (License No. SCXK 2019-0002). All animal experiments were conducted in compliance with the National Institutes of Health Guide for the Care and Use of Laboratory Animals and were approved by the Laboratory Animal Welfare Ethics Committee of the Institute of Radiation Medicine, Chinese Academy of Medical Sciences (Approval No: IRM-DWLL-2024196). The animals were housed under standard conditions (temperature 22 °C ± 2 °C, humidity 40%_70%) with free access to food and water.

### Establishment of bleomycin-induced pulmonary fibrosis model and treatment

Mice were randomly divided into six groups (n = 10): control, model (BLM), three PDT-LD4 groups (60, 120 and 240 μg/kg, intratracheal), and pirfenidone (PFD) group. Pulmonary fibrosis was induced by a single intratracheal instillation of bleomycin (5 mg/kg), whereas control mice received sterile saline. From day 7 post-modeling, mice in the PDT-LD4 groups were administered LD4 (in 100 μL saline) weekly via intratracheal instillation. Thirty minutes after each dose, the thoracic region was irradiated using a 650 nm semiconductor laser (WSLS-650-500 M-200 M-H4; China) with a spot size of 1 cm^2^. The irradiation duration was 1,000 s per mouse to deliver an energy density of 25 J/cm^2^. The laser output power was verified before each experiment using a PM100D power meter (Thorlabs, United States) to ensure stability. The 650 nm wavelength was selected for irradiation as it corresponds to a strong absorption peak of LD4, ensuring efficient activation of the photosensitizer. This PDT protocol was repeated weekly for 3 weeks. On day 22, after a 24-h fast, all mice were euthanized, and lung tissues were collected and fixed in 4% paraformaldehyde for further analysis.

### Measurement of intracellular ROS by flow cytometry

Intracellular reactive oxygen species (ROS) levels were detected using the fluorescent probe 2′,7′-dichlorodihydrofluorescein diacetate (DCFH-DA). After treatments, HELF cells were harvested, washed with pre-warmed phosphate-buffered saline (PBS), and resuspended in PBS at a density of 1 × 10^6^ cells/mL. The cell suspension was then incubated with 10 µM DCFH-DA for 30 min at 37 °C in the dark. Following incubation, cells were washed twice with cold PBS to remove excess probe and resuspended in ice-cold PBS for immediate analysis by flow cytometry. Flow cytometer equipped with a 488 nm excitation laser. Fluorescence emission was collected using a 530/30 nm bandpass filter (FL1 channel). A minimum of 10,000 events per sample were acquired for analysis. Data were analyzed using FlowJo v10.2 software.

### Protein extraction and quantification

For proteomic analysis, the middle lobe of right lung was dissected from each mouse. Tissues from individual mice were processed separately (not pooled) to account for biological variability, with n = 3 per group used for this analysis. Lung tissue were homogenized in 600 μL of ice-cold lysis buffer (8 M urea supplemented with protease inhibitors). The homogenates were subjected to intermittent sonication (1 s on, 2 s off, for a total of 120 s) and subsequently centrifuged at 14,000 × g for 20 min at 4 °C to collect the supernatant. Protein concentration was determined using a bicinchoninic acid (BCA) assay. Protein quality and integrity were assessed by sodium dodecyl sulfate-polyacrylamide gel electrophoresis (SDS-PAGE). Further separation was performed using a RIGOL L-3000 rapid resolution high-performance liquid chromatography (RP-HPLC) system following the manufacturer’s instructions.

### Peptide identification by LC-MS/MS

Lyophilized lung tissue powders were reconstituted in 0.1% formic acid, centrifuged as described above, and 1 μg of protein equivalent was subjected to LC-MS/MS analysis. The acquired raw data were processed using MaxQuant software (version 2.0.3.0) for label-free quantification. Protein identification was carried out by matching spectra against the UniProt database.

### Biochemical assays

Blood samples were obtained from anesthetized mice by enucleation and collected in centrifuge tubes. They were then incubated at room temperature for 30 min to permit complete clotting. The clotted samples were centrifuged at 3,500 rpm for 10 min at 4 °C. Subsequently, the serum was carefully aspirated, aliquoted, and stored at −80 °C until further analysis. Serum levels of interleukin-6 (IL-6; Invitrogen, 88-7064), interleukin-1β (IL-1β; Invitrogen, 88-7013A), and tumor necrosis factor-α (TNF-α; Invitrogen, BMS607-3TEN) were measured using commercial enzyme-linked immunosorbent assay (ELISA) kits. Additionally, the activities or concentrations of myeloperoxidase (MPO; A044-1-1), glutathione (GSH; A006-1-1), malondialdehyde (MDA; A003-1-2), and superoxide dismutase (SOD; A001-1-2) in serum were determined using assay kits from the Nanjing Jiancheng Bioengineering Institute (China), according to the respective manufacturers’ protocols.

### Histopathological analysis

Formalin-fixed lung tissues were embedded in paraffin and sectioned at a thickness of 5 μm. The severity of pulmonary fibrosis was evaluated on Masson’s trichrome-stained sections using the Ashcroft scoring system (Ashcroft et al., 1988). Briefly, at least 10 random fields per section at 100x magnification were scored from 0 (normal lung) to 8 (total fibrotic obliteration). Lung inflammation on H&E-stained sections was scored based on the extent of inflammatory cell infiltration using a semi-quantitative scale from 0 (none) to 4 (severe), as previously described (Wu et al., 2019; Wang et al., 2024; Rong et al., 2021).

### Molecular docking study

The molecular docking procedure was conducted with AutoDock Vina 1.1.2, following the preparation of both the receptor and ligand in AutoDock TooL 1.5.6 and subsequent visualization and mode analysis of the results in PyMOL 1.3. The crystal structure of the target protein MRC1 (PDB ID: 7L65) was retrieved from the Protein Data Bank. The protein structure was preprocessed by adding hydrogen atoms, removing water molecules, and optimizing the hydrogen-bonding network. Docking calculations were performed using default parameters.

### Statistical analysis

Data are expressed as mean ± standard deviation (S.D.). Group comparisons were performed using a two-tailed Student’s t-test for two groups and ordinary one-way analysis of variance (ANOVA) followed by Tukey’s test for multiple groups, respectively.

## Results

### The effect of PDT-LD4 on HELF cells

The synthesis and characterization process of the porphyrin derivative LD4 (Figure 1A) is the same as that described by Meng et al. As determined by the MTT assay, no significant cytotoxicity was observed for LD4 at concentrations up to 240 μg/mL (Figure 1B). Light activation of LD4 (PDT-LD4) resulted in a marked suppression of HELF cells growth across all tested doses in both prophylactic and therapeutic settings, while the black reaction had little to no effect on cell growth (Figures 1C,D).

### PDT-LD4 ameliorated lung inflammation and protected the integrity of the lung in the BLM-induced PF mice

**FIGURE 2:**
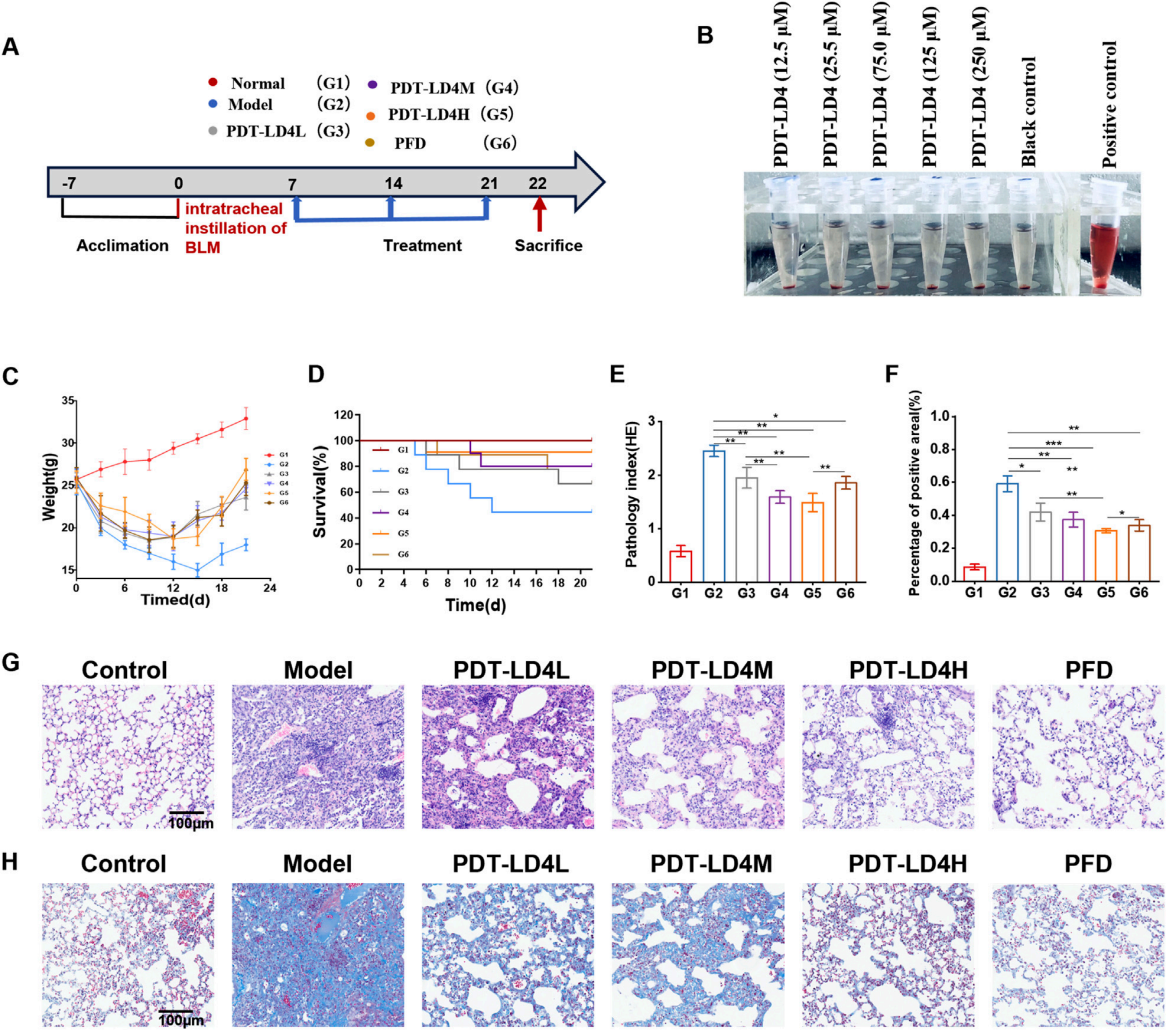
PDT-LD4 ameliorated lung inflammation and protected the integrity of the lung in BLM-induced PF mice **(A)** BLM induced PF schematic diagram. **(B)** Hemolysis rates of RBCs incubated with different concentrations of LD4. **(C)** The body weight. **(D)** Mice mortality rate. **(E,G)** H&E staining of lung tissue and inflammation score; Morphologic and morphometric analyses (×100); **(F,H)** Masson staining of the lung tissues (×100). Data were expressed as mean ± SD (n = 6–10 per group), *P < 0.05, **P < 0.01, ***P < 0.001.

The schematic diagram of animal handling is shown in Figure 2A. LD4 has excellent water solubility, with a hemolysis rate of less than 5% at 120 μg/mL, demonstrating good blood compatibility (Figure 2B). Following model establishment, a marked decrease in average body weight was observed across all bleomycin-administered groups, whereas control animals exhibited a consistent weight gain (Figure 2C). Treatments with PFD and all doses of PDT-LD4 significantly attenuated this BLM-induced weight loss. In line with this, survival outcomes were severely compromised in the BLM group. In stark contrast, intervention with PFD and PDT-LD4 substantially enhanced survival, with the PDT-LD4H group showing the most favorable outcome (Figure 2D).

Examination of HE stained lung sections under light microscopy (Figure 2E) demonstrated clear alveolar architecture and absence of significant inflammation in control animals (Yang et al., 2024). The BLM group exhibited severe inflammatory infiltration, structural distortion, and early fibrotic foci. These pathological changes were markedly ameliorated in both PFD and PDT-LD4 treatment groups. Semiquantitative inflammation scoring confirmed significant higher scores in the BLM group compared to controls, which were effectively reduced by PDT-LD4 treatment in a dose-dependent manner (Figure 2G).

Masson’s trichrome staining (Figure 2F) revealed minimal blue-stained collagen deposition in control lungs. Extensive collagen accumulation was evident within fibrotic foci of BLM-treated mice, which was substantially reduced following PFD or PDT-LD4 intervention. Semiquantitative fibrosis scoring corroborated these observations (Figure 2H), indicating that PDT-LD4 significantly inhibited BLM-induced collagen deposition.

**FIGURE 3:**
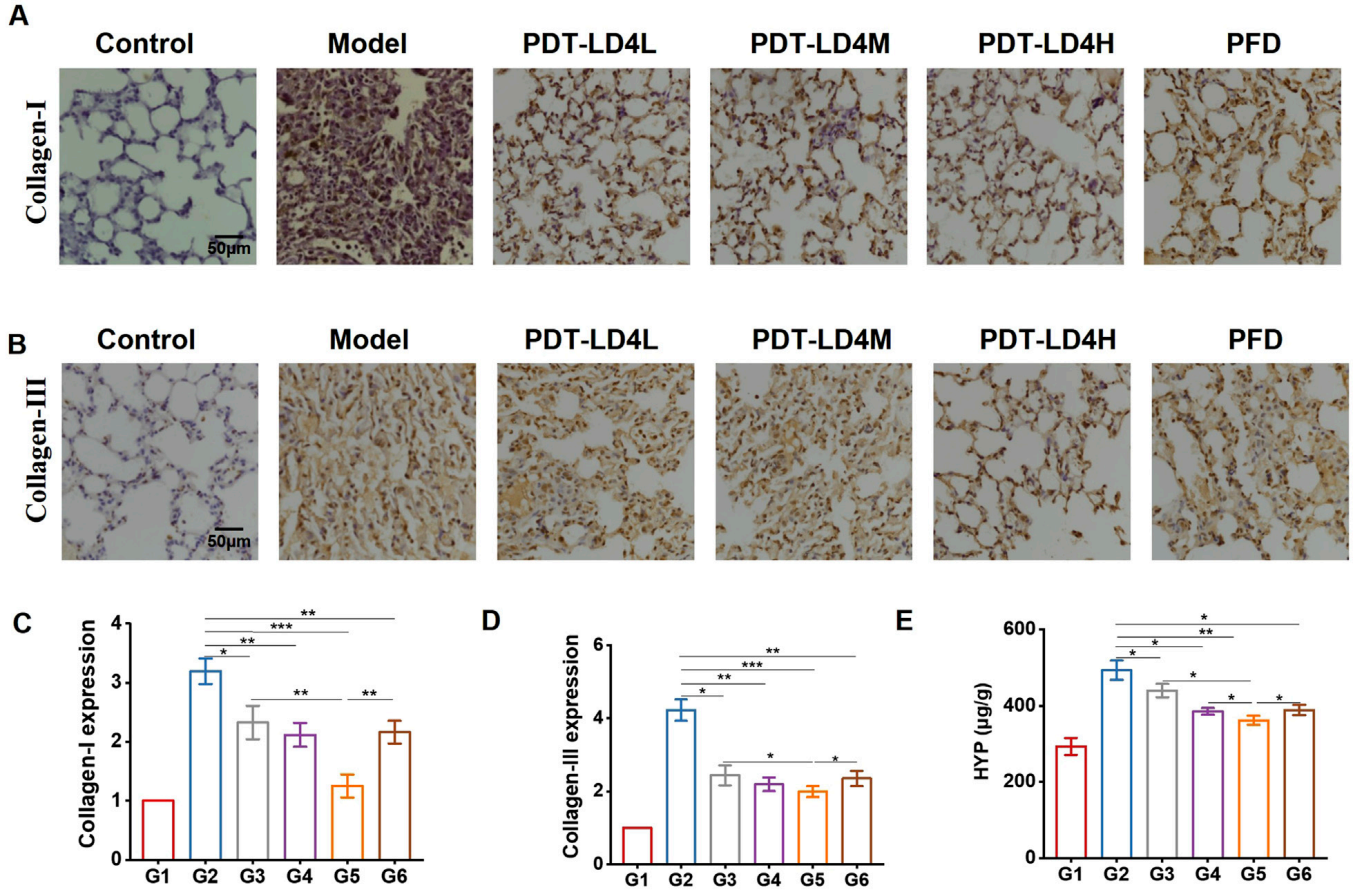
PDT-LD4 alleviated the degree of fibrosis in BLM-induced PF mice. **(A–D)** Collagen I and Collagen III was detected by immunohistochemical staining and analyzed. **(E)** BLM induced to a significantly increase of lung HYP levels (n = 4–5). Data were expressed as mean ± SD, *P < 0.05, **P < 0.01, ***P < 0.001.

Immunohistochemical analysis demonstrated significantly elevated expression of Collagen I and Collagen III in the BLM-group lungs compared to controls. Both PFD and PDT-LD4 treatments markedly reduced the expression of these collagen subtypes (Figures 3A–D), suggesting inhibition of aberrant collagen synthesis. Consistent with these findings, hydroxyproline (HYP) content, a biochemical marker of collagen deposition, was significantly increased in the BLM group relative to controls. All PDT-LD4 dose groups showed significantly lower HYP levels compared to the BLM group (Figure 3F), further confirming the anti-fibrotic efficacy of PDT-LD4.

### PDT-LD4 decreased the expression of inflammatory cytokines in the BLM-induced PF mice

**FIGURE 4:**
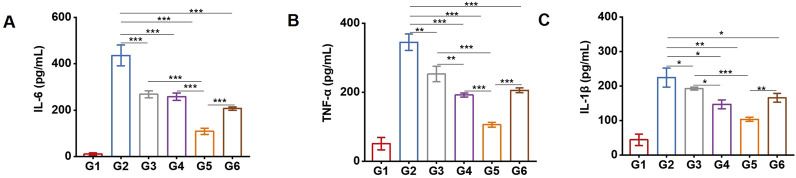
PDT-LD4 decreased the expression of inflammatory cytokines in BLM-induced PF mice. The content of IL-6 **(A)**, TNF-α **(B)** and IL-1β **(C)** levels were tested by ELISA.

The levels of key inflammatory cytokines, including *IL-1β, IL-6,* and *TNF-α*, were measured in serum. As shown in Figures 4A–C, compared with the control group, the model group exhibited significantly elevated concentrations of these cytokines. Both PFD and PDT-LD4 treatments markedly reduced their expression, with PDT-LD4 showing a clear dose-dependent suppression effect.

### PDT-LD4 attenuated oxidative stress in BLM-induced PF mice

**FIGURE 5:**
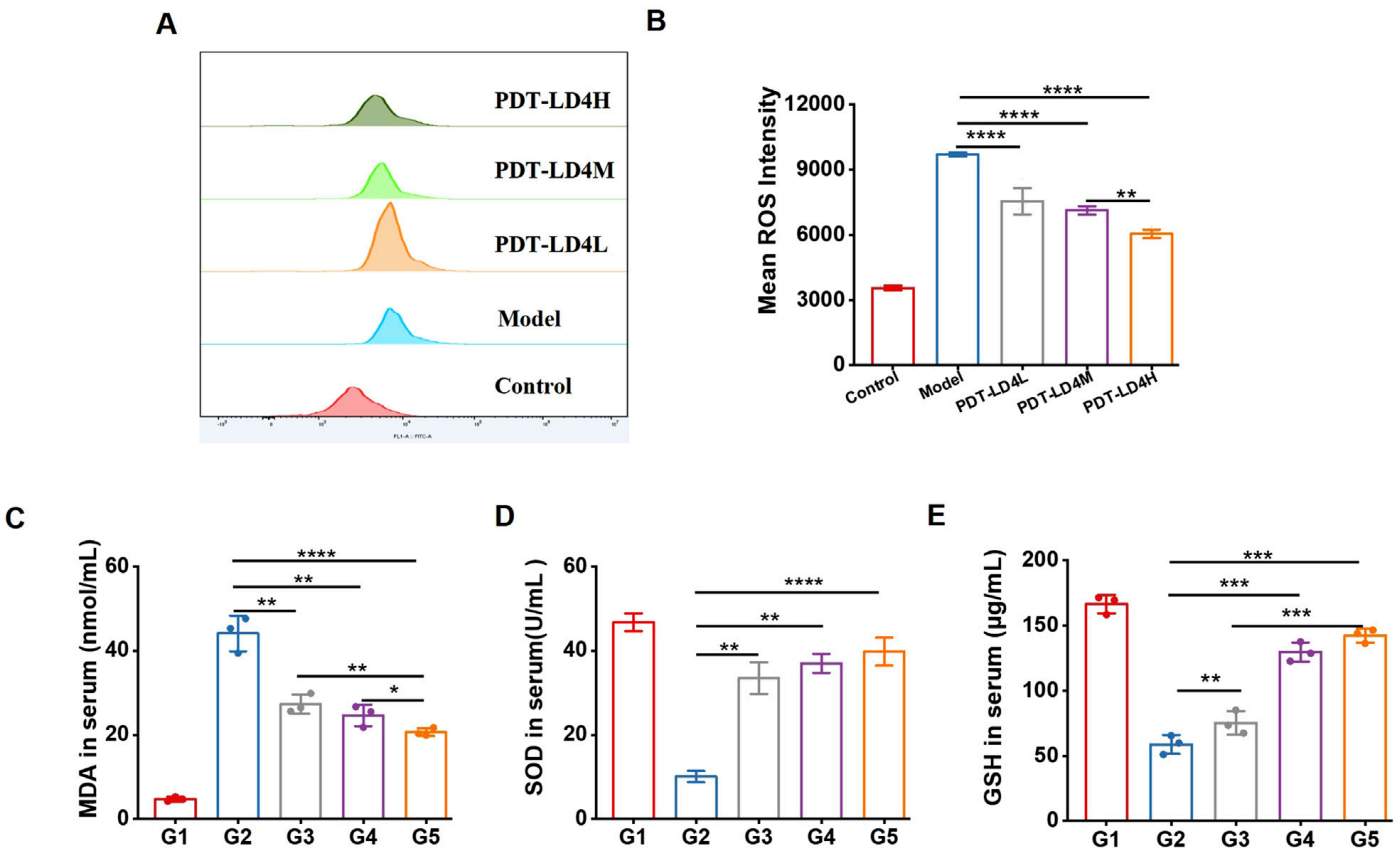
PDT-LD4 attenuated oxidative stress in BLM-induced PF mice. **(A–E)** The content of ROS **(A,B)** in HELF cells, MDA **(C)**, SOD **(D)** and GSH **(E)** levels in mice serum were measured by flow cytometer and ELISA.

Oxidative stress plays a critical role in PF pathogenesis. MDA, a primary product of lipid peroxidation, serves as a key indicator of oxidative stress; its accumulation induces damage to cell membranes and organelles. Measuring MDA levels reflects the extent of lipid peroxidation and indirectly indicates cellular injury. In contrast, GSH and SOD exert antioxidant effects, protecting cells against oxidative damage. We assessed several redox-related factors, including ROS, GSH, SOD, and MDA. As shown in Figures 5A-E, PDT-LD4 suppressed intracellular ROS generation. Serum levels of GSH and SOD, which were higher in the control group, decreased significantly in the BLM group but were markedly restored following PDT-LD4 or PFD treatment. Conversely, MDA content was significantly elevated in the BLM group and reduced after PFD or PDT-LD4 administration. All three drug concentrations exhibited dose-dependent effects. These results collectively indicate that PDT-LD4 effectively alleviates oxidative stress in BLM-induced PF mice.

### PDT-LD4 reprograms the protein profile of lung tissues in the BLM-induced PF mice

**FIGURE 6:**
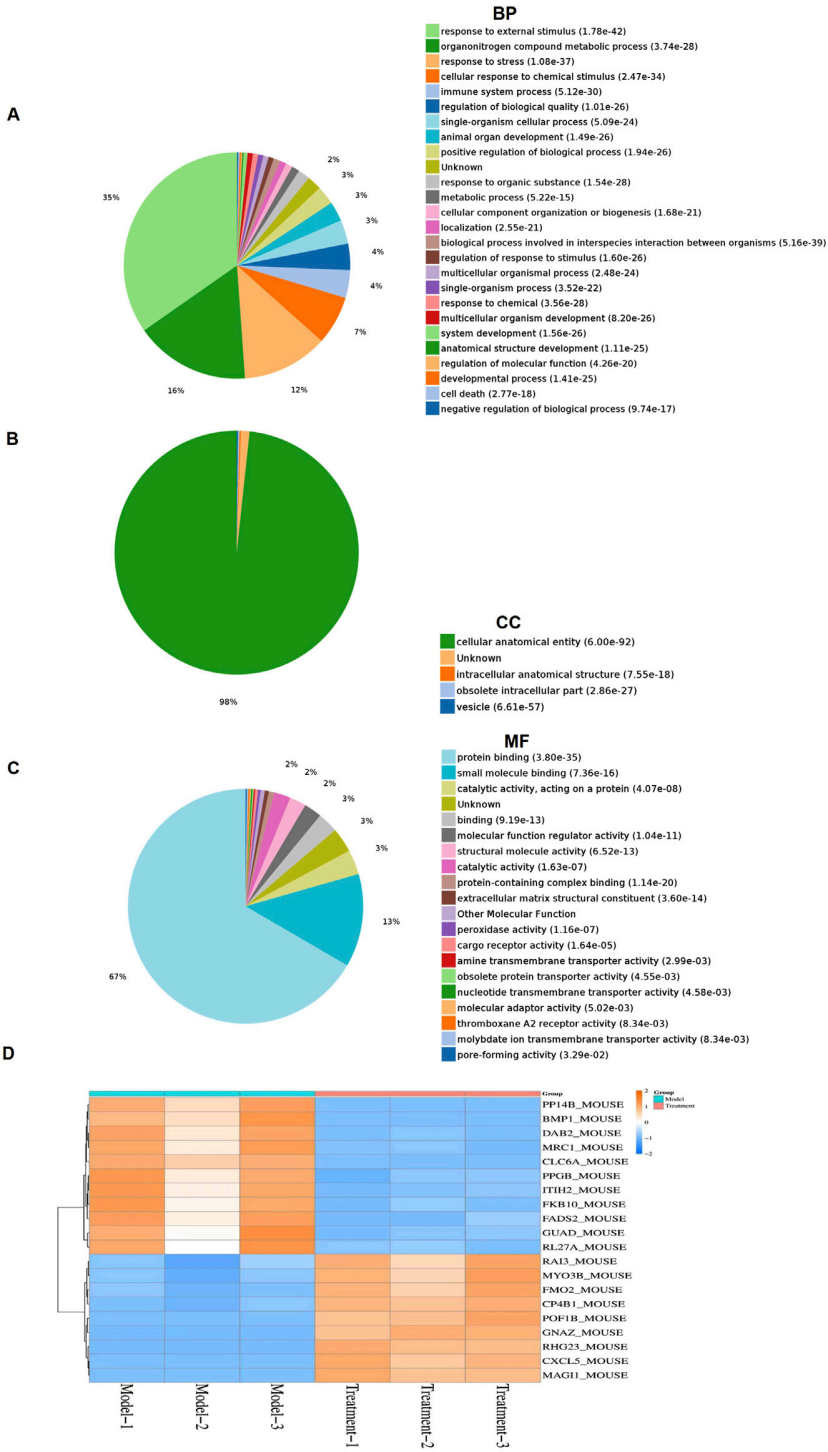
PDT-LD4 reprograms the protein profile of lung tissues in BLM-induced PF mice **(A–C)** All identified proteins were classified according to the first 20 gene ontology ratios sorted by enrichment degree (−log10 [*P-value*]) and protein number according to biological process, cell composition and molecular function. Over-representation analysis was used for functional enrichment analysis, and the t-test based on hypergeometric distribution. **(D)** Heat map of 40 differentially expressed proteins among the model and PDT-LD4 groups. Each column represented a sample, and each row represented a factor; red indicated elevated protein expression, green indicated decreased protein expression.

To investigate the mechanism of PDT-LD4 in treating PF, we performed a label-free quantitative proteomic analysis of lung from mice in the control, BLM-induced model, and PDT-LD4H treatment groups. Differentially expressed proteins (DEPs) were identified using a t-test, with significance thresholds set at *P *≤ 0.05 and a fold change ≥1.5. Gene Ontology (GO) enrichment analysis revealed that significantly related to organonitrogen compound metabolic process and biological proces. GO analysis of biological processes showed that differentially expressed proteins between the control and BLM model groups were significantly related to organonitrogen compound metabolic process and biological process involved in interspecies interaction between organisms (Supplementary Figure S1A). However, treatment with PDT-LD4 changed the protein expression profiles (Figure 6A). In terms of cellular component, the differentially expressed proteins were related to cellular anatomical entity and extracellular vesicle (Supplementary Figure S1B), indicating that treatment with PDT-LD4 resulted in changes in intracellular anatomical structure (Figure 6B). In terms of molecular function, the differentially expressed proteins were mainly derived from protein binding and small molecule binding (Supplementary Figure S1C), while treatment with PDT-LD4 changed the catalytic activity, acting on a protein (Figure 4C). We screened 20 significantly different proteins using heat maps to illustrate the differing protein expression profiles in the lung samples. The results showed that expression of macrophage mannose receptor (*MRC1*)in model mice was obviously increased (Supplementary Figure S2D), while this upregulation was eliminated following treatment with PDT-LD4 (Figure 4C). *MRC1* plays a crucial role in the body’s process of recognizing and eliminating pathogens such as bacteria and viruses, so we selected *MRC1* for further study. A heatmap of the top 20 significantly altered proteins showed that *MRC1* expression was substantially upregulated in model mice (Supplementary Figure S2D), which was effectively reversed by PDT-LD4 treatment (Figure 4C). Given the crucial role of *MRC1* in pathogen recognition and clearance, it was selected for further investigation.

### Molecular docking study

**FIGURE 7:**
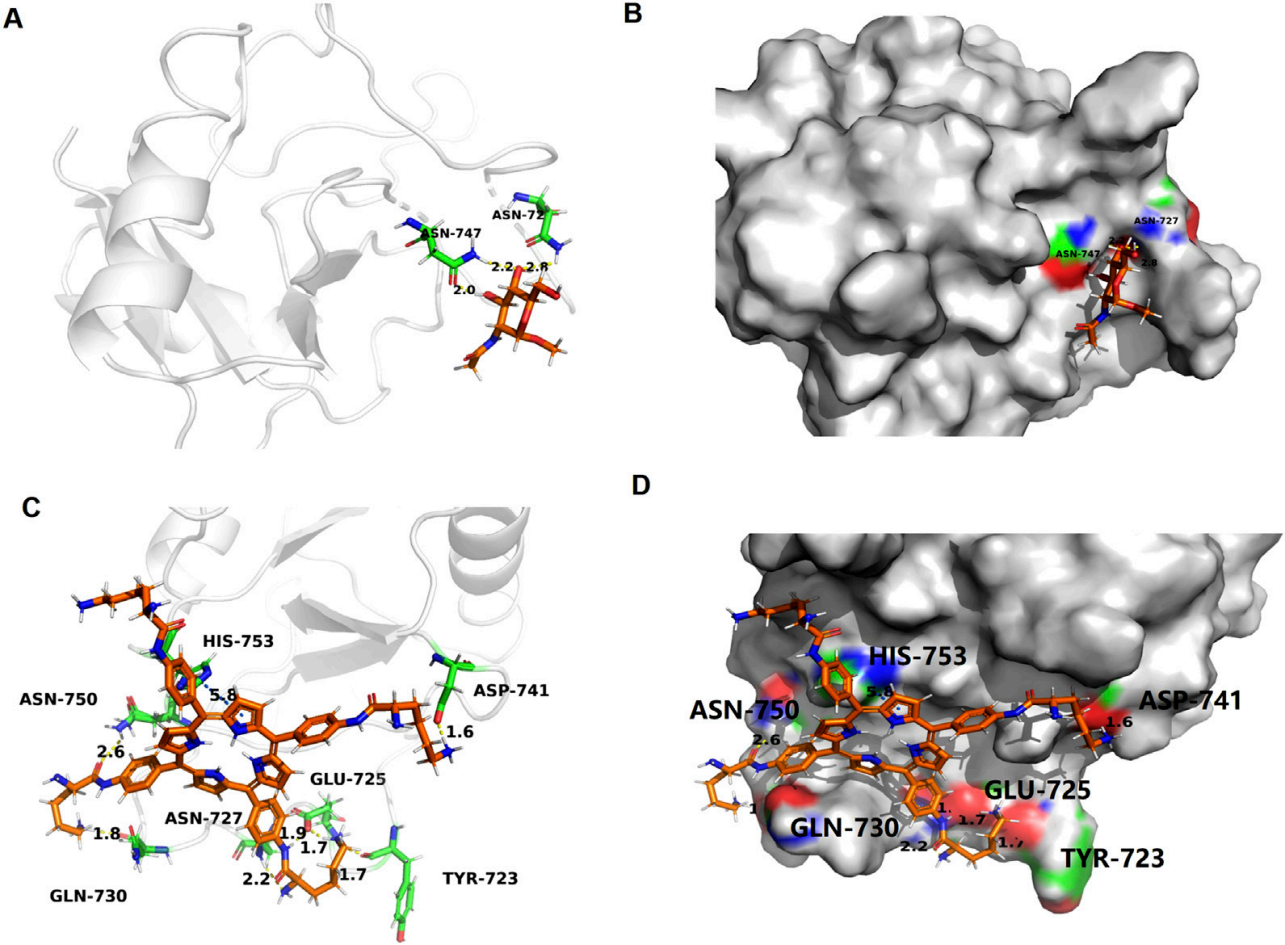
Docking and binding pattern into MRC1 active site for ligand **(A,B)** and LD_4_ **(C,D)**. Hydrogen bonds - yellow lines, π-π bonds - blue lines.

To elucidate the potential molecular interaction between LD4 and the *MRC1,* molecular docking simulations were performed. The crystal structure of *MRC1* (PDB ID: 7L65) was retrieved from the Protein Data Bank and used as the receptor model. As visualized in Figures 7C,D, LD4 was successfully docked into the binding pocket of the 7L65 protein. The binding mode analysis revealed that LD4 engages with the active site primarily through the formation of hydrogen bonds (yellow), π-π stacking interactions (blue), and contacts with specific amino acid residues (orange). The stability of the LD4-7L65 complex was assessed by calculating the binding energy. A more negative binding energy value indicates a more stable and favorable interaction. As summarized in Supplementary Table S1, the docking score and computed binding energy for LD4 were superior to those of the native ligand, suggesting that LD4 may form a more stable complex with *MRC1* and potentially exhibit a stronger binding affinity. Molecular docking analysis revealed that LD4 engages with the binding pocket of *MRC1* through specific interactions with key amino acid residues. Specifically, LD4 forms hydrogen bonds with TYR-723, GLU-725, ASN-727, GLN-730, ASP-741, ASN-750 and participates in π–π stacking interactions with HIS-753.

### Safety assessment of PDT-LD4 treatment

**FIGURE 8:**
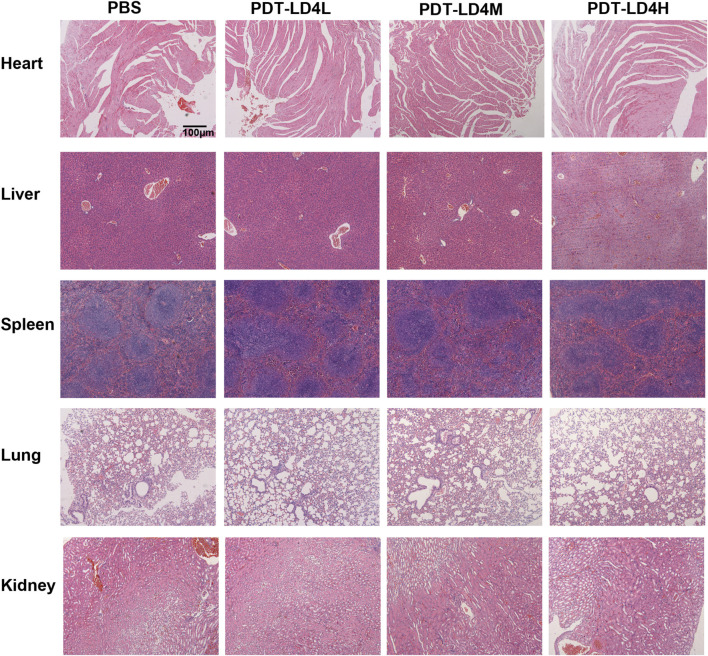
Safety assessment of PDT-LD4 treatment.

To evaluate the potential systemic toxicity of PDT-LD4, a comprehensive histopathological assessment was performed. Mice were randomly allocated into four groups: a normal control group and three treatment groups receiving low-, medium-, and high-dose PDT-LD4 (60, 120, and 240 μg/kg, respectively). Following the completion of the treatment regimen, major organs, including the heart, liver, spleen, lungs, and kidneys, were harvested for histological examination. Representative HE stained sections from each group were examined by light microscopy. As illustrated in Figure 8, the histological architecture of all examined organs appeared normal and well-preserved across all groups. In conclusion, the results demonstrate that PDT-LD4, even at the highest dose employed, does not induce observable systemic toxicity or damage to major organs, supporting its favorable safety profile for further therapeutic investigation.

## Discussion

Pulmonary fibrosis (PF) is a progressive and often fatal interstitial lung disease characterized by excessive deposition of extracellular matrix, for which current therapeutic options remain limited to slowing rather than halting or reversing disease progression (Kim et al., 2025; Sharma et al., 2025). Our study demonstrates that LD4-mediated photodynamic therapy (PDT) confers a significant protective effect against bleomycin-induced PF in mice. The improvement in survival and the attenuation of weight loss, classic indicators of overall disease burden in PF model, were strongly supported by histopathological evidence. The marked reductions in inflammation scores and collagen deposition, as visualized by H&E and Masson’s trichrome staining respectively, confirm that PDT-LD4 effectively interrupts the core pathological progression of fibrotic lesion formation.

The pathogenesis of PF is intrinsically linked to a dysregulated inflammatory response (Mehta et al., 2021). Our finding that PDT-LD4 significantly reduced the levels of key pro-inflammatory cytokines (*IL-1β, IL-6*, and *TNF-α*) aligns with the known role of these mediators in driving fibroblast proliferation and activation. The observed anti-inflammatory effect is consistent with previous reports on the immunomodulatory capacity of PDT in other disease models (Panagiotidis et al., 2025) and suggests that modulating the inflammatory microenvironment is a crucial mechanism by which PDT-LD4 exerts its anti-fibrotic action. This multi-cytokine suppression may offer an advantage over approaches targeting single pathways. Oxidative stress is a well-established driver of PF, contributing to epithelial cell injury and fibroblast activation (Zhang et al., 2025b). The efficacy of PDT-LD4 in restoring the levels of key antioxidants (*GSH* and *SOD*) while reducing markers of oxidative damage (*MDA* and *ROS*) indicates a potent capacity to rebalance redox homeostasis. This is particularly significant, as oxidative stress persists throughout the progression of PF (Yao et al., 2025). The demonstrated antioxidant effect provides a mechanistic foundation for the observed cellular protection and supports the exploration of PDT as a strategy to mitigate oxidative damage in fibrotic diseases.

The ultimate hallmark of PF is the aberrant accumulation of collagen and other ECM components (Yao et al., 2025). Our data provide compelling biochemical evidence that PDT-LD4 directly targets this fibrotic endpoint. The downregulation of Collagen I and III expression, coupled with the significant reduction in hydroxyproline content, confirms that the therapy effectively inhibits the core process of ECM deposition. This anti-fibrotic effect is central to its therapeutic potential, as reversing ECM accumulation is a major goal of anti-fibrotic drug development.

*MRC1* is associated with anti-inflammatory (M2) macrophage polarization, which can influence tissue repair and fibrosis (Liu et al., 2024; Liang et al., 2025; Wan et al., 2025). The reversal of BLM-induced *MRC1* upregulation by PDT-LD4 suggests a role in modulating macrophage phenotype, adding an immunomodulatory dimension to its mechanism. The molecular docking studies, showing a stable and favorable binding interaction between LD4 and *MRC1*, provide a structural hypothesis for this observed biological effect. This finding opens a new avenue for understanding how PDT can influence the immune microenvironment in fibrosis. To bridge the gap between the proteomic identification of *MRC1* and the multifaceted phenotypic outcomes, we propose a mechanistic hypothesis informed by emerging insights into macrophage biology and immunometabolism. Our data, which show BLM-induced *MRC1* upregulation that is reversed by PDT-LD4, align with a model where MRC1 is not merely a marker but a functional component within a pro-fibrotic signaling network. A foundational study by von Ehr et al. established a clear antagonistic relationship between *TGF-β* signaling and MRC1, demonstrating that TGF-β1 downregulates *Mrc1* in microglia, whereas silencing TGF-β signaling robustly upregulates it (Von Ehr et al., 2020). This suggests that the *MRC1* upregulation in our BLM model may reflect a dysregulation of *TGF-β* signaling, a known driver of fibrosis. Furthermore, Liu et al. (Liu et al., 2024) recently demonstrated that deficiency in NLRX1, a mitochondrial immune regulator, promotes M2 macrophage polarization with concomitant increases in*MRC1*expression and *TGF-β* secretion, leading to exacerbated renal fibrosis. Crucially, this pro-fibrotic phenotype was driven by a metabolic shift towards enhanced mitochondrial oxidative phosphorylation (OXPHOS), underscoring the intimate link between macrophage metabolism, polarization, and fibrotic output. Integrating these findings, we hypothesize that PDT-LD4, potentially through direct interaction with *MRC1*, disrupts a pro-fibrotic feedback loop. This intervention may work by modulating macrophage immunometabolism, potentially shifting the balance away from the OXPHOS-dependent M2 state. The subsequent downregulation of MRC1 and a shift in macrophage polarization would then lead to a reduction in the secretion of pivotal pro-fibrotic and pro-inflammatory cytokines, including *TGF-β*, *IL-1β*, and *IL-6*. The attenuation of this inflammatory milieu, coupled with a direct impact on redox balance, would explain the observed alleviation of oxidative stress. The culmination of these events reduced inflammation, oxidative stress, and profibrotic signaling provides a coherent mechanistic basis for the significant inhibition of collagen deposition we observed. This integrative model, which connects PDT-LD4 binding to *MRC1*, subsequent immunometabolism reprogramming of macrophages, and the resolution of key fibrotic pathways, offers a robust and multi-tiered framework to explain the anti-inflammatory, antioxidant, and anti-fibrotic efficacy of our treatment.

The role of *MRC1* and M2 macrophages in fibrosis is complex and context-dependent. While the M2 phenotype is traditionally associated with anti-inflammatory and tissue repair functions, persistent and dysregulated M2 activation is now widely recognized as a key driver of pathological fibrosis in conditions like PF. In this pro-fibrotic context, M2 macrophages, often characterized by high *MRC1* expression, produce excessive levels of profibrotic mediators such as TGF-β, PDGF, and galectins, which directly promote fibroblast proliferation and collagen deposition. Therefore, the BLM-induced upregulation of *MRC1* observed in our model likely signifies a shift towards a pro-fibrotic macrophage phenotype. Consequently, the reversal of this upregulation by PDT-LD4 may represent a normalization of the macrophage response, attenuating its contribution to the fibrotic cascade rather than suppressing a beneficial anti-inflammatory process. It is important to note that the current study lacks functional validation (e.g., via *MRC1* knockdown or blockade) to definitively establish its causal role in the therapeutic effects of PDT-LD4. Therefore, while our data position *MRC1* as a promising target, its precise role as a key mediator remains to be confirmed in future investigations. The connection between proteomics (*MRC1*) and the main phenotypic results is weak. The study does not effectively bridge how targeting *MRC1* mechanistically leads to the observed anti-inflammatory, antioxidant, and anti-fibrotic effects. The translational potential of any new therapy hinges on its safety profile. While numerous experimental anti-fibrotic strategies focus on inhibiting single pathways, such as specific cytokine signaling or fibrotic kinases, their efficacy is often limited by the complex, multi-factorial nature of PF pathogenesis. In contrast, PDT-LD4 demonstrates a superior and more comprehensive therapeutic profile by concurrently targeting several core pathological processes. It not only directly suppresses the aberrant activation of fibroblasts and collagen deposition but also effectively alleviates the chronic inflammatory response and rebalances oxidative stress. This multifaceted, synergistic mechanism of action enables PDT-LD4 to disrupt the vicious cycle of fibrosis at multiple nodes, thereby offering a potentially more robust and effective intervention compared to mono-mechanistic approaches. The molecular docking results suggest a stable binding mode between LD4 and *MRC1*. However, these computational predictions require experimental validation, such as surface plasmon resonance (SPR) to determine binding affinity or site-directed mutagenesis of the identified key residues (e.g., Asp-105, Tyr-265) to confirm their necessity for the interaction. The absence of such functional validation is a limitation of the current study, and these experiments form a critical part of our future work. Our comprehensive histopathological assessment of major organs revealed no significant toxicity associated with PDT-LD4, even at the highest dose tested. This favorable safety profile, consistent with previous reports on LD4, is a critical strength and supports its further development. The dose-dependent effects observed across multiple parameters underscore its pharmacological relevance.

In conclusion, our results position PDT-LD4 as a promising multi-targeted therapeutic candidate for PF. It simultaneously addresses inflammation, oxidative stress, and collagen deposition—key pathological features of the disease. The identification of *MRC1* as a potential target offers a novel direction for mechanistic research. Future studies should focus on elucidating the precise signaling pathways involved, particularly the *in vivo* role of *MRC1* modulation, and validating these findings in additional experimental models or longer-term studies to firmly establish its therapeutic promise and advance toward clinical application. From a translational perspective, the intratracheal administration route used in this study mirrors feasible clinical delivery methods for targeting pulmonary diseases, enhancing the potential of PDT-LD4 for future development as a localized therapy for PF patients.

## Data Availability

The original contributions presented in the study are publicly available. This data can be found here: https://www.iprox.cn/page/project.html, submission number PXD070823.
