# Supplementary material for: Photodynamic therapy with a novel photosensitizer inhibits BLM-induced pulmonary fibrosis in mice via MRC1-mediated pathway
Source: Front Pharmacol. 2025 Nov 27;16:1714450. doi: 10.3389/fphar.2025.1714450 (PMC12695786; doi:10.3389/fphar.2025.1714450)
Supplement: Supplementary file 1 [file DataSheet1.docx]

Photodynamic therapy with a novel photosensitizer inhibits BLM-induced pulmonary fibrosis in mice via MRC1-mediated pathway^.^

Fig1

A


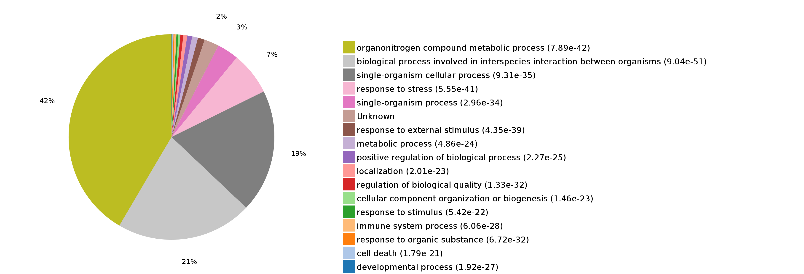


B


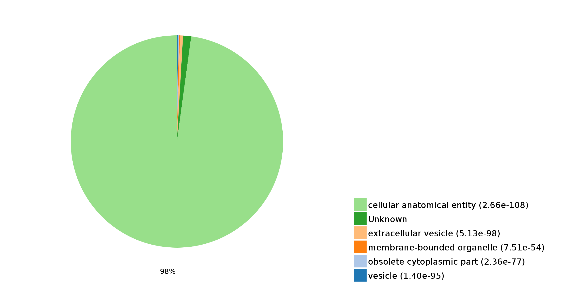


C


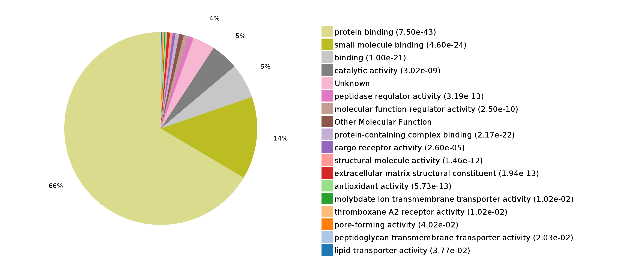


D


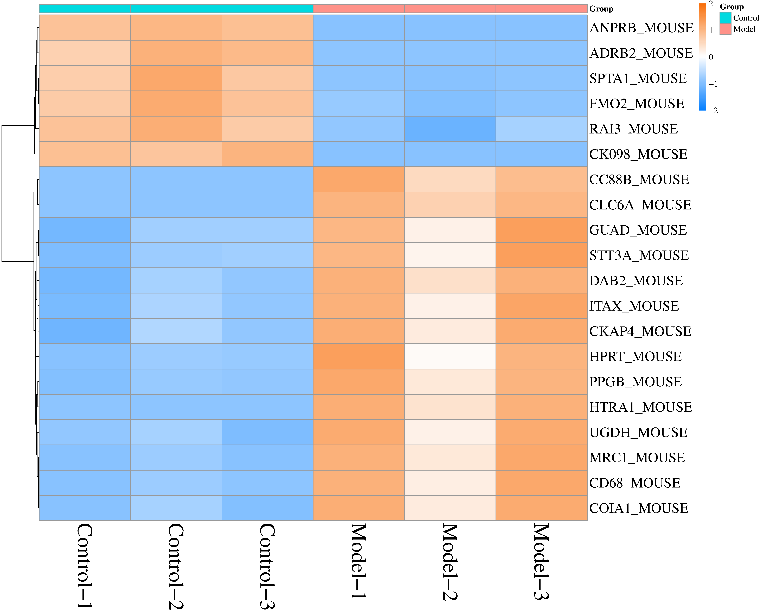


**Supplementary Tables**

**Supplementary Table S1**

Table 1 Molecular docking of MRC1 and LD4

| Compound | Docking score(kcal/mol) | | Binding energy(kcal/mol) | |
| --- | --- | --- | --- | --- |
| Ligand | | -4.564 | | -35.655 |
| LD_4_ | | -7.066 | | -93.674 |

**
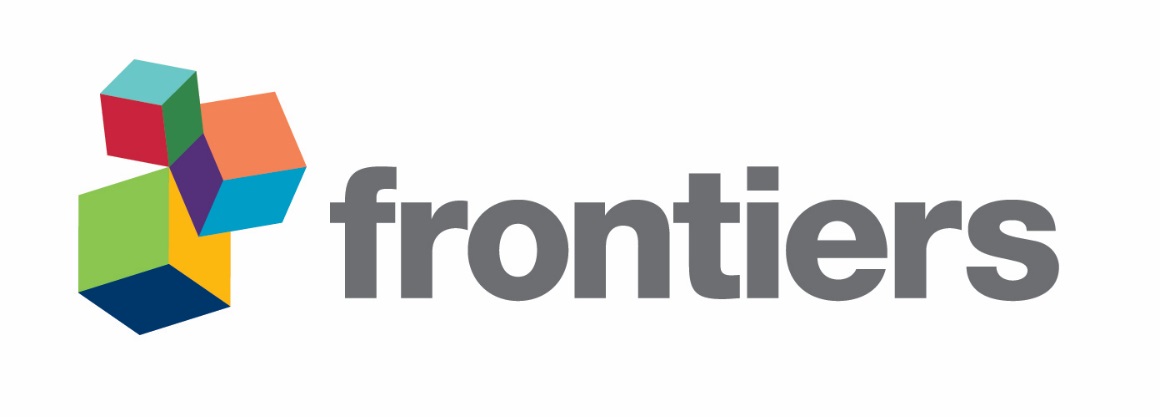
**
